# Case Reports: Transformation of End-Stage Neuroendocrine Tumors With Uncontrollable Liver Metastasis Into a Novel or Additional Functional Phenotype

**DOI:** 10.3389/fonc.2020.555963

**Published:** 2020-09-25

**Authors:** Takaomi Kessoku, Noritoshi Kobayashi, Masato Yoneda, Yuki Kasai, Anna Ozaki, Naoki Okubo, Michihiro Iwaki, Takashi Kobayashi, Tsutomu Yoshihara, Yusuke Kurita, Yasushi Honda, Motohiko Tokuhisa, Hiroto Ishiki, Takashi Hibiya, Satoshi Fujii, Atsushi Nakajima, Yasushi Ichikawa

**Affiliations:** ^1^Department of Palliative Medicine, Yokohama City University Hospital, Yokohama, Japan; ^2^Department of Gastroenterology and Hepatology, Yokohama City University Graduate School of Medicine, Yokohama, Japan; ^3^Department of Oncology, Yokohama City University Hospital, Yokohama, Japan; ^4^Department of Palliative Medicine, National Cancer Center Hospital, Tokyo, Japan; ^5^Department of Pathology, Yokohama City University Hospital, Yokohama, Japan; ^6^Department of Molecular Pathology, Yokohama City University Graduate School of Medicine, Yokohama, Japan

**Keywords:** neuroendocrine tumor, insulinoma, gastrinoma, liver metasatasis, transformation

## Abstract

**Background:** Neuroendocrine tumors (NETs) are rare, but their worldwide incidence is gradually increasing. NETs are generally heterogeneous; however, in rare cases, they have been shown to change their phenotype (i.e., nonfunctional to functional or one functional phenotype to the addition of another functional phenotype). Here, we present two cases of liver metastatic NETs with phenotype transformation at the advanced stage that led to life-threatening events.

**Case presentation:** A 73-year-old woman had a small intestinal nonfunctional NET with liver metastasis. After uncontrollable liver metastasis at the advanced stage, she developed duodenal perforation with hypergastremia. The patient was treated with octreotide and proton pump inhibitors and underwent endoscopic closure for duodenal perforation, but her general condition gradually deteriorated, and she died 2 weeks after duodenal perforation. Another patient, a 50-year-old man, had a functional NET (gastrinoma) with liver metastasis and duodenal ulcer. After uncontrollable liver metastasis at the advanced stage, he developed hypoglycemia. Although octoreotide and diazoxide were administrated for hyperalimentation, his hypoglycemia was uncontrollable, and he died after 4 months owing to general deterioration.

**Conclusion:** The present cases show that advanced NETs with treatment-uncontrollable liver metastasis can transform their phenotype, specifically from a nonfunctional NET into a functional NET, and from one functional NET into the addition of another functional NET. These experiences suggest that the presence of treatment-resistant liver metastasis might be a hallmark of the potential to gain novel functions.

## Introduction

Neuroendocrine tumors (NETs) are rare, but their worldwide incidence is gradually increasing (1). There has been a particular increase in the incidence of nonfunctional NETs, and most patients present with metastatic disease (1). The majority of NETs are indolent and asymptomatic at early stages, but medical intervention is necessary to control many symptoms derived from tumor volume and hormone secretion at advanced stages (2). NETs are generally heterogeneous, but in rare cases nonfunctional NETs change their phenotype into functional tumors (3, 4). We describe two cases of liver metastatic NETs with phenotype transformation at the advanced stage that led to life-threatening events.

## Case Description

### Case 1

**Figure 1:**
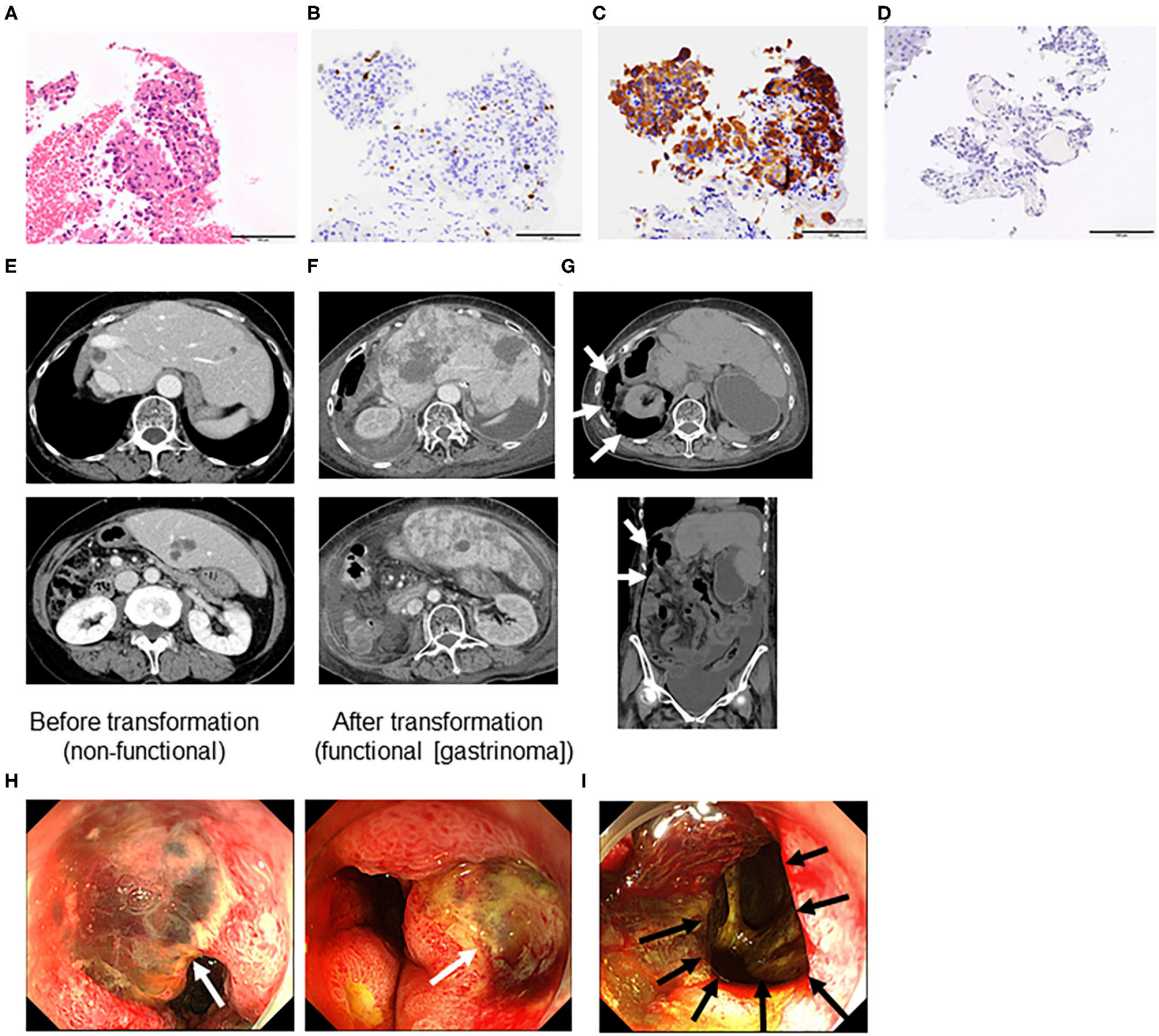
Microscopic features assessed by liver biopsy before transformation **(A–D)**, computed tomography **(E–G)**, and endoscopy **(H,I)** of a grade 2 neuroendocrine tumor (Case 1). **(A)** Hematoxylin and eosin staining. Immunohistochemistry of **(B)** Ki-67, **(C)** synaptophysin, and **(D)** gastrin. (All ×40 magnification). **(E)** Liver metastasis before transformation (non-functional neuroendocrine tumor [NET]), **(F)** liver metastasis after transformation into a functional NET [gastrinoma], and **(G)** duodenal perforation indicated by retroperitoneal free air (arrows). **(H)** Multiple duodenal ulcers (white arrows) and **(I)** perforation hole toward the retroperitoneum (black arrow).

A 73-year-old Japanese woman complained of right upper abdominal pain in 2003. She visited a high-volume center and was diagnosed with a small intestinal nonfunctional carcinoid tumor with liver metastasis (grade 2) based on clinical and histological findings (Figures 1A–D). She received partial small intestinal resection and right lobe hepatectomy in June 2003.

### Case 2

**Figure 2:**
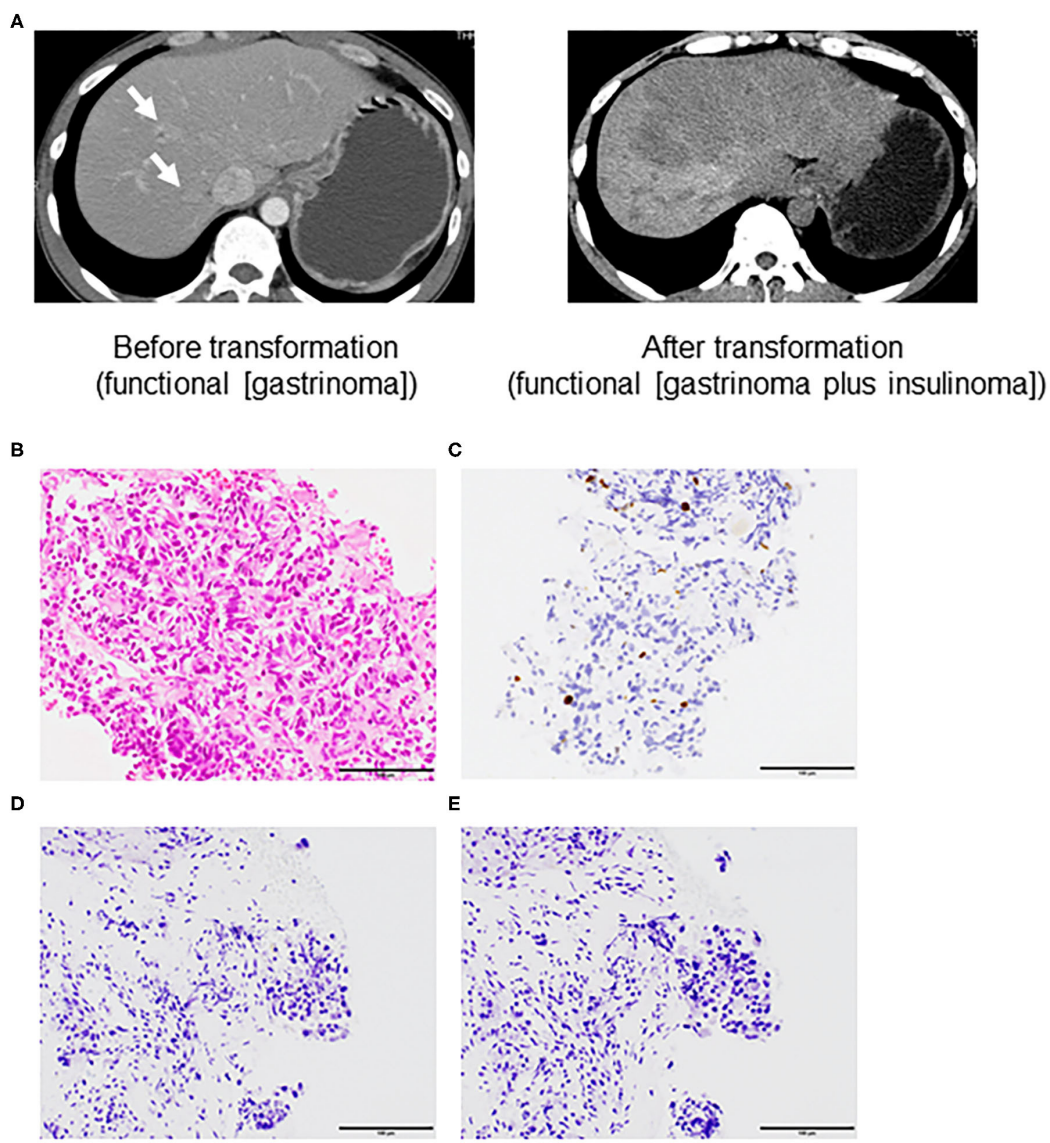
Computed tomography **(A)** and microscopic features assessed by liver biopsy before transformation **(B–E)** of a grade 2 neuroendocrine tumor (Case 2). **(A)** left panel; liver metastasis (arrow) before transformation (functional neuroendocrine tumor [NET; gastrinoma]) and right panel; liver metastasis after transformation into the addition of another functional NET [gastrinoma and insulinoma]. **(B)** Hematoxylin and eosin staining. Immunohistochemistry of **(C)** Ki-67, **(D)** gastrin, and **(E)** insulin (All ×40 magnification).

A 50-year-old man was admitted to the local hospital for diarrhea and vomiting in May 2010. Computed tomography revealed a large tumor in the pancreas body and tail (maximum diameter, 60 mm) and multiple liver tumors (maximum diameter 20 mm) (Figure 2A, left panel). Gastrointestinal endoscopy revealed severe gastritis, and his serum gastrin level was elevated. We diagnosed him with pancreatic NET (grade 2, Ki-67 3.7%) with liver metastasis (gastrinoma) based on liver biopsy specimens and clinical findings (Figures 2B,C). Immunohistochemistry analysis further revealed gastrin and insulin negativity in the tumor (Figures 2D,E).

## Diagnostic Assessment

### Case 1

The patient experienced recurrence of her liver tumors and underwent segmental liver resection and transarterial chemoembolization. However, multiple liver tumors continued to grow (Figure 1E). Therefore, she received octreotide from November 2014 to April 2015. She visited our hospital in April 2015, and we performed octreoscan in May 2015, which revealed uptake in multiple liver tumors and lymph nodes. Therefore, she received three rounds of peptide receptor radionuclide therapy (PRRT) (July 2015, October 2015, and January 2016). Her liver and lymph node lesions gradually decreased, so we deemed her to have achieved partial response in 2017. However, computed tomography findings in March 2018 revealed multiple liver and lung tumors, as well as metastatic lymph nodes. Octreoscan indicated moderate to intense uptake in multiple liver tumors and lymph nodes. We performed re-PRRT (June and August 2018). Due to disease progression (increased liver metastasis), we performed ultrasound-guided liver biopsy, which led to a diagnosis of NET (G2, Ki-67 3.4%). We administered everolimus but had to discontinue treatment due to disease progression. Therefore, the treatment was changed to streptozocin. However, after 7 days of streptozocin, the patient was admitted to our hospital complaining of abdominal pain. She had elevated serum gastrin levels (1,903 pg/mL, normal range = 42–200 pg/mL, Table 1). Additionally, computed tomography and endoscopy showed increased liver metastasis (Figure 1F) and perforation of multiple duodenal ulcers (Figures 1G–I). The patient was treated with octreotide and anti-acid medication (proton pump inhibitors) and underwent endoscopic closure for duodenal perforation, but her general condition gradually deteriorated, and she died 2 weeks after the duodenal perforation.

**Table 1:** Laboratory findings before and after transformation in Case 1.

**Serum parameter**	**Normal range**	**Before transformation**	**After transformation**
Gastrin (pg/mL)	42–200	–	1,903
Amylase (U/L)	44-132	67	54
TSH (μIU/mL)	0.5-5.0	1.49	1.74
FT3 (pg/mL)	2.3-4.0	2.65	3.15
FT4 (pg/mL)	0.9-1.7	1.51	1.7

*FT; free triiodothyronine TSH; thyroid-stimulating hormone*.

### Case 2

The patient received 10 courses of chemotherapy (etoposide and cisplatin) from August 2010 to May 2011 without severe adverse events. We also performed transarterial chemoembolization from the common hepatic artery in November 2010. He received octreotide from August 2010 to July 2012. The pancreatic tumors showed gradual progression (maximum size, 10 mm) and invaded the portal vein. Therefore, we also administered the mammalian target of rapamycin (mTOR) inhibitor (everolimus) beginning in February 2012. We evaluated the pancreas and liver tumors in May 2012. The pancreas tumor was 7 cm with 3 cm portal invasion, and there were seven liver tumors (maximum size, 20 mm). We deemed that everolimus was effective but not sufficient to cause tumor shrinkage and prolong the patient's survival. Therefore, we changed his treatment to PRRT and sunitinib, but this was discontinued after he experienced multiple duodenal ulcers, duodenal perforation, and exacerbation of the liver metastasis (Figure 2A, right panel). He was admitted to the emergency room with a sudden loss of consciousness, and his blood glucose level was 46 mg/dL during transport. The patient regained consciousness after the intravenous infusion of glucose solution. At that time, his fasting blood glucose level was 38 mg/dL, C-peptide level was 6.4 ng/mL, and insulin level was 50.3 μIU/mL (Table 2). Elevated fasting insulin and C-peptide levels relative to glucose levels suggested that the hypoglycemia was due to hyperinsulinemia. Although octoreotide and diazoxide were administered for hyperalimentation, his hypoglycemia was uncontrollable. After 4 months, he died due to general deterioration.

**Table 2:** Laboratory findings before and after transformation in Case 2.

**Serum parameter**	**Normal range**	**Before transformation**	**After transformation**
FBS (mg/dL)	60–90	101	38
Insulin (μIU/mL)	1.8–12.2	–	50.3
C-peptide (ng/dL)	1.1–4.4	–	6.4
Gastrin (pg/mL)	42–200	5,915	5,270
Amylase (U/L)	44–132	70	54

*FBS; fasting blood sugar*.

## Discussion

In the present study, we described two rare cases of treatment-uncontrollable liver metastatic NETs. One was a nonfunctional small intestinal NET with liver metastasis and the other was a functional pancreatic NET (pNET; gastrinoma) with liver metastasis that underwent phenotypic transformation to a functional NET (gastrinoma) and an insulinoma, respectively, which eventually led to death. PNETs are divided into nonfunctional and functional NETs; nonfunctional NETs are far more common. Functional NETs are further subdivided based on the specific hormone secreted, including insulinomas, gastrinomas, glucagonomas, and VIPomas, among others. Although rare, the ability of pNETs to acquire new functional phenotypes has been previously reported (4). One study reported that non-functional NETs were transformed into insulinomas that cause repeated hypoglycemic episodes due to insulin hypersecretion (3). In our first case, non-functional small intestine, which is not a pNET, was transformed into a functional NET (gastrinoma). Owing to the rarity of this phenomenon, little is known about the mechanisms by which tumors transform into gastrinomas or insulinomas or gain function. The origin of NETs might be related to the type of hormone secreted, such as gastrin or insulin. In view of these cases, it is important that patients with end-stage nonfunctional NETs with expanding liver metastases be administered anti-acid medications such as proton pump inhibitors to prevent peptic ulcer after diagnosis of functional NETs such as gastrinoma.

Our second case was initially diagnosed as a functional pNET (gastrinoma) with treatment-uncontrolled liver metastasis, and it transformed into the addition of another functional NET (gastrinoma and insulinoma). Three previous case reports (five cases) have described the transformation of nonfunctional pNETs into functional pNETs (insulinoma) (3–5). However, this is the first report to describe a functional NET secreting gastrin and insulin. Therefore, advanced-stage NETs might be able to gain additional functions, allowing for the secretion of additional hormones.

Refractory hypoglycemia is an important cause of morbidity and mortality in patients with NETs and contributes to poor prognosis (3, 4). Due to the rarity of patients with functionally altered pNETs, data on the treatment of these patients are scarce. Therefore, treatment choices are limited to rare case reports or expert opinions. Systemic therapies (somatostatin analogs, tyrosine kinase inhibitors such as sunitinib, mTOR inhibitors such as everolimus, and PRRT) and liver-targeted therapies (e.g., transarterial chemoembolization) have been used in the treatment of nonfunctional pNETs with insulinoma transformation. However, none of these therapies have proven to be highly effective in these cases.

Lee et al. (6) previously presented a case of sunitinib-induced hypoglycemia occurring in a patient with nonfunctional pancreatic neuroendocrine carcinoma that had metastasized to the liver. In this case, endogenous hyperinsulinemia was observed, but insulin production by tumor cells was not evaluated because hypoglycemia improved with small doses of prednisolone. A recent report describes a similar rare case of transformation of a nonfunctional NET into an insulin-producing tumor, which, similar to the present case, metastasized to the liver (3). However, the hypoglycemia could not be attributed to sunitinib treatment, as various chemotherapy regimens were being used to treat the patient. In our case, severe hypoglycemia developed after treatment with sunitinib and thyroid hormones alone, but it has not been reported to induce hypoglycemia. These cases resemble nonfunctional pancreatic neuroendocrine carcinomas with liver metastases, suggesting that there may be a common mechanism, such as hepatic tissue environment. These reports also suggest the possibility of drug-induced transformation.

Interestingly, all previously published cases of NET transformation have been associated with hepatic metastasis, suggesting a drug-induced transformation or a role of the intrahepatic microenvironment in this process (3, 4). However, recent data in pituitary neuroendocrine cells suggest the presence of pluripotent progenitor cells that may be involved in the plasticity of the tumor phenotype (7). Both the cases, we experienced had high levels of liver function markers (i.e., aspartate transaminase and alanine aminotransferase) after transformation. However, it is unclear whether the elevated levels of liver function markers were caused by drug treatment or systemic deterioration. Although a subpopulation of clinically silent insulin-producing cells may become prominent in the liver, drug-induced transformation cannot be excluded from the clinical course.

The strengths of our report are the rarity of these cases and the importance of this phenomenon. By increasing the general awareness of this phenomenon, we believe that patients with NETs complicated by liver metastases can be managed appropriately. The limitations of this report are that (1) there are only two cases, (2) histological finding does not show the endocrine production (gastrin) before transformation in case 2, and (3) we lack histological assessment suggesting gastrin and insulin production in tumor cells at the time of transformation. Therefore, there is no direct evidence that the phenotype was caused by the tumor cells. Additionally, it is unclear whether the transformation was caused by environmental factors or whether it was drug-induced.

## Conclusion

These cases and a review of the literature suggest that advanced-stage NETs with treatment-uncontrolled liver metastasis can transform from a nonfunctional NET into a functional NET or from one functional phenotype into the addition of another functional phenotype. Considering the possibility of such a serious life-threatening event, we recommend that oncologists and palliative medical doctors carefully monitor abdominal symptoms and hypoglycemic symptoms. Importantly, patients with nonfunctional NETs with end-stage disease and expanding liver metastasis need to take an anti-acid medication such as proton pump inhibitors to prevent peptic ulcers in the event of transformation into a functional NET, such as gastrinoma. More cases need to be accumulated to elucidate the mechanism of transformation, which is important for prevention.

## Patient Perspective

In this case study, the patients were involved in the conduct of the study. The development of the research question was particularly based on the patients' experiences. The results of this study will be disseminated by the international report to patients and medical staff.

## Ethics Statement

The studies involving human participants were reviewed and approved by Yokohama City University. The patients/participants provided their written informed consent to participate in this study. Written informed consent was obtained from the individual(s) for the publication of any potentially identifiable images or data included in this article.

## Author Contributions

TKe and NK participated in study design. TKe, NK, MY, YKa, AO, NO, MI, TKo, TY, YKu, YH, MT, HI, TH, SF, AN, and YI participated in interpretation.

## Conflict of Interest

The authors declare that the research was conducted in the absence of any commercial or financial relationships that could be construed as a potential conflict of interest.

## Abbreviations

NET: neuroendocrine tumor
PRRT: peptide receptor radionuclide therapy
pNET: pancreatic neuroendocrine tumor
mTOR: mammalian target of rapamycin.
